# Reviewers acknowledgement

**DOI:** 10.1002/2211-5463.13733

**Published:** 2023-12-06

The editors of *FEBS Open Bio* would like to thank all those who have given their time and expertise to review articles submitted for publication in Volume 13. Although *FEBS Open Bio* does not seek to judge the importance of submissions, our reviewers carefully scrutinize the experimental design and results of all papers and challenge authors about their conclusions.

The names of these reviewers are listed below; we apologize if we have inadvertently omitted anyone.

Eslam Abbass

Walaa Abdel Wahab

Alka Aggarwal

Rodrigo Aguiar

Manuel H. Aguiar‐Oliveira

Dinesh Ahirwar

L.E. Alvares

Héctor M. Alvarez

Adriano Angelucci

Franca Anglani

Jenniffer Angulo

David M. Ansell

Andrea Armirotti

Simon Arthur

Anoop Arunagiri

Mark P. Ashe

Yasmeen Attia

Massimo Aureli

Matías Antonio Ávila Zaragozá

Tudor Constantin Badea

Michael Bader

Luis Alberto Baena‐Lopez

Angshuman Bagchi

Shairaz Baksh

Luigi Balasco

Kiran Bali

Ivan Ballesteros

Yoshio Bando

Hiroko Bannai

Lee Bardwell

Rebecca Barnes

Rafał Bartoszewski

Reetobrata Basu

Zsófia Bata

Christine Belloir

Elena Bencurova

Mohammed Bensellam

Dharmendra K. Bhargava

Aditi Bhargava

Qing Bi

Claudie Bian

Oliver Bischof

István Bojti

Usa Boonyuen

Didier Boquet

Christopher Borcuk

Guney Boso

Alessio Branchini

Laura Brunnthaler

Boudewijn M. T. Burgering

Peter Burkovics

Stefano Cacchione

C. Joaquin Caceres

Sebastien Cagnol

Gaetano Cairo

Philip C. Calder

Laura Calvo

Ilaria Canobbio

Raphael Carapito

Daniela Carlisi

John P. Carr

Daniel D. Carson

Ronaldo Carvalho Araujo

Giorgio Caserta

Filipe Castro

Marjorie Cepeda‐Plaza

Sohini Chakraborty

Joydeep Chakraborty

Kun‐Che Chang

Marika Charalambous

Manish Charan

Ligong Chen

Huaiyong Chen

Gongxing Chen

John Chilton

Elena Chiricozzi

Gopal Chovatyia

David J. Clark

Richard Clarkson

Rosemary Clyne

Steven L. Cobb

Eric Coïc

Federica Comella

Anne Cooke

Erico T. Costa

James Costello

Marie Couturier

Nancy Craig

Zoran Culig

Dimitra Dafou

Thomas Dandekar

Undurti N. Das

Judith Davie

Yoshiharu Deguchi

Giovanna Della Porta

Selami Demirci

Jeroen den Hertog

Evgeny V. Denisov

Carsten Deppermann

Giovanni Di Bernardo

Alessio Di Fonzo

Ian M. C. Dixon

José Clóvis Jr. do Prado

Camila O. Dos Santos

Gregory R. Dressler

Yang Duan

M. Beatriz Durán‐Alonso

Yves Durocher

Amit Dutt

Nicolas Dzamko

Adrienne Edkins

Noriyuki Egawa

Sigrun Eick

Andrey Elchaninov

Sherien M. El‐Daly

Mohammad ElGamacy

Fumito Endo

Yasushi Enokido

Young Woo Eom

Tomas Erban

Abdul Samath Ethayathulla

Isabel Fabregat

Fulvia Farabegoli

Camille Faure

Maria Fazzari

Julia Fedotova

Eva Fenyvesi

Julian Fernandez

Timothy Florin

Dimy Fluyau

Alessandro Formaggioni

Samuel Fountain

Heather Francis

Jing Fu

Xing Fu

Masato Fujioka

Mikako Fujita

Atsunori Fukuhara

Tsuyoshi Fukushima

Satoru Funamoto

Koichi Furukawa

Luca Galluzi

Yong‐Gui Gao

Ning Gao

Lin Gao

Carmen C. Garcia

Haitao Ge

Robert Gelfand

Maria Genander

Chiara Gentili

Róna Gergely

Michele Ghidini

Reena Ghildyal

Nady Golestaneh

Saúl Gómez‐Manzo

Yoichi Gondo

Xiaohua Gong

Mirian Angelene González Ayón

Marina Govoroun

Oliver Griesbeck

Henri Gruffat

Malgorzata Grzesiak

Guoqiang Gu

Lingyu Guan

Gamze Guney Eskiler

Dongsheng Guo

Mansour Haddad

Zhen Han

Xavier Hanoulle

John W. Hanrahan

Azizul Haque

Joshua M. Hare

Markus Hartl

Takeshi Hase

Kazuhiko Hashimoto

Sanchita Hati

Diane Hatton

Yoshiki Hayashi

Armagan Hayirli

Elke H. Heiss

Rob Henning

Leanna Hernandez

Angel Herráez

Riley Hess

Tim Hewitson

Dominique Heymann

Mark A. Hink

Rita Hirmondó

Hidekazu Hiroaki

Kyle L. Hoehn

Jörg Höhfeld

Hermann‐Georg Holzhütter

Koichi Honke

Tamarisk Kerry Horne

Gabriel Hornink

Gabriel Horta‐Baas

Keiko Hosohata

Chao‐Kai Hsu

Yingbo Huang

Bo Huang

Pin Huang

Benjamin D. Humphreys

Iskander M. Ibrahim

Shingo Ichimiya

Jin‐ichi Inokuchi

Hisamitsu Ishihara

Fumitoshi Ishino

Tohru Itoh

Takeshi Itoh

Ivan Ivanov

Kazuhisa Iwabuchi

Luis Izquierdo

Conxita Jacobs‐Cachá

Jyoti Jaiswal

Trinath Jamma

Eric Jan

Sujata Jana

Elisa Helena Farias Jandrey

Anand D. Jeyasekharan

Jan Jezek

Rajesh Kumar Jha

Stefanie Jonas

Michael Jonz

Ute Jungwirth

Yasutomi Kamei

Mohammad Kamran

Masashi Kanai

Oguz Kanca

Renuka Kandhaya‐Pillai

Yu Kanesaki

Shinichi Kano

Kensaku Kasuga

Vladimir Katanaev

Vincent P. Kelly

Megan Keniry

Krishna Mohan Ketha

Bilon Khambu

Kum Kum Khanna

Mahmoud Mohamed Khattab

Amit Khot

Keekwang Kim

Seyun Kim

Wanil Kim

Chandra Kishore

Nancy Klauber‐DeMore

Elena Klimova

Krista Kobylianskii

Weijia Kong

Nont Kosaisawe

Yutaka Koyama

Kanako Koyanagi

Anne Krapp

Donald Kuhn

Janesh Kumar

Rajender Kumar

Ashwini Kumar Ray

Sharda Kumari

Ursula Kummer

Ozgur Kutuk

Sowmya Lakshmi

Yu‐Yu Lan

Gerald Larrouy‐Maumus

Fabien Le Grand

Rozenn Le Hir

Robert Ledeen

Etienne Lefal

Yun Lei

Suvi‐Katri Leivonen

Roger Leng

Olivia Lenoir

Guillermo León

Martina Lepore Signorile

Gaetano Leto

Zhenfei Li

Mingli Li

Lanlan Li

Yen‐Liang Li

Jian‐Xin Li

Sara Liin

Benedikt Linder

Jonathan A. Lindquist

Fei Liu

Zhengshuai Liu

Gang Liu

Murielle Lombard

Bruno Lomonte

Svenja Lorenz

Martin K. Lotz

Zhao‐huan Lou

Peter Low

Kang Lu

Yongping Lu

James Lu

Giuseppe Lucarelli

Anastasios Lymperopoulos

Qingjun Ma

Harold MacGillavry

Peter Macheroux

Mateusz Maciejczyk

Luca Madaro

Sergio Madrigal‐Carballo

Stephanie Maia Acuña

Wojciech Makalowski

Koyeli Mapa

Tarunendu Mapder

Margherita Maranesi

Maurilia Marcacci

Luis Antonio Mariñas‐Pardo

Alvaro Marín‐Hernández

Mija Marinković

Edileusa Cristina Marques Gerhardt

Sandra L. Martin

Kenneth Martin

Alfredo Martinez

Jonathan Martinez‐Fabregas

Claudia Martini

Dzmitry Matsiukevich

Toshiro Matsui

Alexander V. Mazin

Kirill E. Medvedev

Sabine Middendorp

Gabriella Milan

Akira Mima

Kitti Mintál

Shohei Mitani

Ammar Mohammed AL‐Farga

Zsófia Molnár

Mariana P. Monteiro

Kyle Moore

David Moreau

Ryuichi Morishita

Ana M. Moura‐da‐Silva

Simone Mozzachiodi

Charlotte Muir

Leonhard Müllauer

Enrique Muro

Tatsuro Mutoh

Hossein Naderi‐Manesh

Gergely Nándor Nagy

Nikolett Nagy

Yuji Naito

Shigeki Nakagome

Ikuhiko Nakase

Jun Nakayama

Ki‐Hoan Nam

Vigneshwaran Namasivayam

Asuka Nanbo

Heinz Peter Nasheuer

Carlo Natale

Sangeeta Nath

Manasa Nayak

Joachim Neumann

Xiao Ni

Paramasivam Nithyanand

Wolfgang Nitschke

G.D. Norata

Kinga Nyiri

Rod O'Connor

Keiko Ogawa

Chika Ogura

Shigeru Okada

Toshiro Okazaki

Rui Oliveira

Gustavo Olivos‐Ramirez

José Luis Olloqui Sariego

Heidi Olzscha

Jasminka Omerovic

Nicola Opallo

Tadayuki Oshima

Henrik Oster

Lado Otrin

Petar Ozretić

Balagopal Pai

Vitantonio Pantaleo

Giovanni Paolino

Nazareno Paolocci

Romain Parent

Abbas Parham

Alberto Pascual

Bolay Paul

Evgeny V. Pavlov

Milena Paw

Nancy Pelaez

Antti Pemmari

Andrea Pepper

Timothy R. Peterson

Michael Pierce

Fabien Pierrel

Sergej Pirkmajer

Francesco Pisani

Hidde L. Ploegh

Lejla Pojskic

Indira D. Pokkunuri

Sandra Postić

Evan Pratt

Benjamin Price

Daniel Max Raben

Gergely Attila Rácz

Mehak Rafiq

Fatemeh Rahbarizadeh

Muhammad Farooq Rai

Gábor Rákhely

Gayatri Ramakrishna

Joao Ramalho‐Santos

Vanessa Ramirez

Pablo Ranea‐Robles

Paul A. Reynolds

N. Rhaleb

Engy Risha

Hector Riveros‐Rosas

Jean‐David Rochaix

Christian E. Rocheleau

Oscar Rojas Carrillo

Jorge Luis Rosas‐Trigueros

Ryan D. Ross

Paula Rozo‐Lopez

Yuri Rubtsov

Carolina Ruivo

Remo Castro Russo

Stephen Rutherford

Islam M. Saadeldin

Maja Sabol

Daisuke Saito

Takashi Saito

Afrah Fatthi Salama

Andrés Sánchez Brenes

Federica Sangiuolo

Kumar Singh Sanjay

Prasanna Kumar Santhekadur

Janine Hertzog Santos

Webster Santos

Gerardo Santos‐López

Leandro Sastre Garzón

Wolfgang Sattler

Maximilian Scheer

Augusto Schmidt

Bohdan Schneider

Anthony Schwacha

Harald Schwalbe

Graham Scott

Yusuke Sekine

Sudip Sen

Diane C. Shakes

Mehdi Sharifi Tabar

Ashu Sharma

Atsuyoshi Shimada

Ginga Shimakawa

Osamu Shimmi

Tsuyoshi Shirai

Khawar Siddiqui

Constantine Simintiras

Arjun Singh

Abhishek Singharoy

Weerachai Singhatanadgit

Maria Kløjgaard Skytthe

Alexandre Smirnov

Jim T. Smith

Thierry Soldati

Maria Solesio

H. Song

Maurizio Sorice

Magdalena Sowa‐Kućma

Charles Specht

Alexis Stamatikos

Robin Stanley

Guillaume St‐Jean

Victor Strelstsov

Cheng‐Huang Su

Lakshmi Subhadradevi

Venkat Subramanian

Zhen Sun

Bing Sun

Isaac Kirubakaran Sundar

Supaporn Suttamanatwong

Tetsufumi Takahashi

Masato Tamura

Jalal Taneera

Hailin Tang

A. I. Tarasov

Peter ten Dijke

Suman S. Thakur

Pierre Therizols

Gabriele Toietta

Vjekoslav Tomaic

Tsukasa Tominari

Taisuke Tomita

Signe Torekov

Howard Trachtman

Hiroyuki Tsuchiya

Benjamin Turk

Bettina Ughy

Felix Urra

Andrea Vaiana

Priit Väljamäe

Willem J. H. van Berkel

Folkert van Werven

Máté Varga

Gabriel Vargas

Héctor Vázquez‐Meza

Viktor Vedelek

Nicole Salazar Velmeshev

Massimo Venditti

Patricia Veras

Balázs Veres

Marija Vidovic

Susanne Voelkel

Elmar Wahle

Hong Wan

Lipeng Wang

Jiangxin Wang

Tian‐Li Wang

Zhen Wang

Yan‐jun Wang

Xinghua Wang

Zeneng Wang

Peter A. Ward

Christine J. Watson

Mark Watson

Helen Watson

Thomas S. Weiss

Ora Weisz

Emily Welby

Anna Wendt

Xiaocheng Weng

Andrew Westwell

Blake Wiedenheft

Iain B. H. Wilson

Gordana Wozniak Knopp

Louwrance P. Wright

Yuan Seng Wu

Dong Xiao

Xue Xiao

Hanchu Xiong

Wei‐Hua Xu

Jiake Xu

Shiwen Xu

Kang Xu

Sosuke Yagishita

Atsuko Yamashita

Michiaki Yamashita

Song Yang

Shi‐Bing Yang

Zhentao Yang

Masato Yano

Darrin York

Michael Zahn

Daniel Zajuc

Xingxing Zang

Maria Ines Zanor

Yanhui Zhang

Qiong Zhang

Jian Zhang

Junyu Zhang

Shanfeng Zhang

Tuanjie Zhao

Peng Zhao

Fei Zhao

Yun‐Wen Zheng

Rongjia Zhou

Xinzhou Zhu

Angela Ziebell

Tali Zitman‐Gal

